# Screening by Clinical Breast Examination in Western Kenya: Who Comes?

**DOI:** 10.1200/JGO.2015.000687

**Published:** 2016-01-27

**Authors:** Naftali Wisindi Busakhala, Fredrick Asirwa Chite, Juddy Wachira, Violet Naanyu, Job Wapangana Kisuya, Alfred Keter, Ann Mwangi, Evanjeline Njiru, David Chumba, Lugaria Lumarai, Penina Biwott, Ivan Kiplimo, Grieven Otieno, Gabriel Kigen, Patrick Loehrer, Thomus Inui

**Affiliations:** **Naftali Wisindi Busakhala, Fredrick Asirwa Chite, Juddy Wachira, Violet Naanyu, Ann Mwangi, Evanjeline Njiru, David Chumba, Gabriel Kigen,** and **Thomus Inui,** Moi University School of Medicine; **Lugaria Lumarai** and **Penina Biwott,** Moi Teaching and Referral Hospital; **Job Wapangana Kisuya, Ivan Kiplimo,** and **Grieven Otieno,** Academic Model Providing Access to Healthcare (AMPATH) Oncology; **Alfred Keter** and **Ann Mwangi,** AMPATH Statistics, Eldoret, Kenya; **Patrick Loehrer Sr,** Indiana University Simon Cancer Center; and **Thomus Inui,** Regenstrief Institute, Indianapolis, IN.

## Abstract

**Purpose:**

More than 80% of women with breast cancer in Kenya present to medical care with established late-stage disease. We sought to understand why women might not participate in breast cancer screening when it is offered by comparing the views of a cohort of those who attended a screening special event with those of community controls who did not attend.

**Methods:**

All residents living close to three health centers in western Kenya were invited to participate in screening. Participants (attendees) underwent clinical breast examination by trained physician oncologists. In addition, women who consented were interviewed by using a modified Breast Cancer Awareness Module questionnaire. Nonattendees were interviewed in their homes the following day.

**Results:**

A total of 1,511 attendees (1,238 women and 273 men) and 467 nonattendee women participated in the study. Compared with nonattendees, the women attendees were older, more often employed, knew that breast cancer presented as a lump, and were more likely to have previously felt a lump in a breast. In addition, they were more likely to report previously participating in screening activities, were more likely to have performed breast self-examination, and were less concerned about wasting a doctor’s time. Almost all those surveyed (attendees and nonattendees) expressed interest in future breast cancer screening opportunities.

**Conclusion:**

The women who volunteer for breast cancer screening in western Kenya are more aware of breast cancer than those who do not volunteer. Screening recruitment should seek to close these knowledge gaps to increase participation.

## INTRODUCTION

Breast cancer is the most common cancer in Kenya, accounting for approximately 23% of all cancers in the country.^1^ Kenya is not alone in having such a breast cancer burden. Worldwide, almost 50% of breast cancer cases and 58% of deaths occur in low- and middle-income countries (LMICs).^2^ Breast cancer affects younger women and is more clinically aggressive in women from sub-Saharan Africa than among women from North America.^3^ Screening for breast cancer is important because this disease has a preclinical phase during which the condition is localized and asymptomatic, a stage in which this cancer may have a greater chance of being cured and may have longer survival.^4^ Perhaps because there are low levels of breast cancer awareness and there is limited access to health care, more than 80% of women in western Kenya present late, at a stage associated with poor outcomes.^5,6^

### Screening for Breast Cancer

Mammographic screening of women for breast cancer is widely used in countries in which screening facilities are available.^7,8^ By contrast, because of the lack of access to mammography screening in the public sector, Kenya National Guidelines for cancer management recommend breast self-examination (BSE) and clinical breast examination (CBE) for early cancer detection.^9^

### Efficacy of CBE as a Screening Strategy

Clinical trials of breast cancer screening have reported sensitivity and specificity for CBE screening of 51.7% and 94.3%, respectively.^10-12^ Preliminary results of a cluster-randomized controlled trial in India that used CBE suggested that this approach may uncover significantly more early-stage cancers in the screened population (18.8 per 100,000 women) than emerged in a control group (8.1 per 100,000 women).^10^

### Screening for Breast Cancer in Resource-Constrained Settings

In most LMICs, screening events use CBE and are usually donor-funded special opportunities. In some of these events, most of the abnormalities identified by the clinicians are already symptomatic. The participants in such events may come there to confirm what they already know or to seek further care for an existing disease, not the ideal situation for screening and early detection efforts. This might be one explanation of why screening activities in LMICs find cancers in advanced stages more often than do similar activities in the developed world.^13^ Hoping that we might find ways to encourage women to participate in screening at an earlier stage in their disease natural history, we searched the literature for relevant studies but found no research describing predictors of women’s participation in breast cancer screening in Kenya. For this reason, we conducted a prospective cohort study to ascertain what distinguished women who chose to participate in CBE screening from those who did not participate in our setting. We believed that this kind of information might subsequently be used to design public education efforts that would motivate nonattendees to become attendees.

## METHODS

### Study Site

The Academic Model Providing Access to Healthcare (AMPATH) is a collaboration among Moi University School of Medicine, Moi Teaching and Referral Hospital (MTRH), Kenya’s Ministry of Health, and North American academic institutions led by Indiana University School of Medicine.^14^ Initially, AMPATH focused on HIV care, but it has now broadened its services to include primary health care and chronic disease management, including prevention and care for cancer through the AMPATH Oncology Institute (AOI). Since 2006, annual screening has been performed during the month of October (Breast Cancer Awareness Month). The Walther Project, in which this study is nested, was initiated in 2011 under the auspices of AOI with a grant from Walther Cancer Foundation of Indianapolis, IN, in support of research on cancer prevention in Kenya.

**Fig 1:**
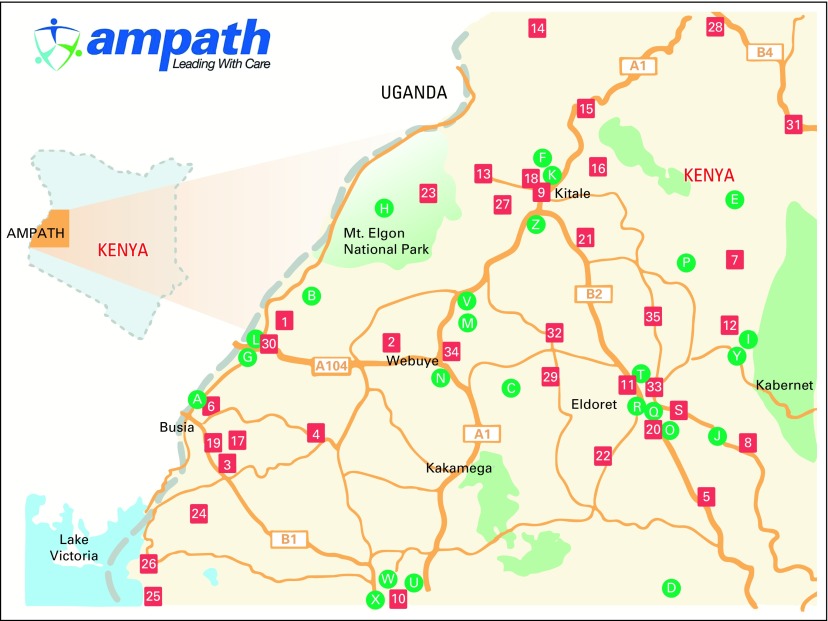
Academic Model Providing Access to Healthcare (AMPATH) catchment area. Reproduced with permission of AMPATH.

During October and November 2012, the Walther Project organized breast health education and screening activities in three communities within the AMPATH catchment area (Fig 1). The study communities were chosen on the basis of unpublished data from the Eldoret Cancer Registry in an attempt to represent counties with high, medium, and low burdens of breast cancer. Uasin Gishu, Nandi, and Mount Elgon account for 45%, 5%, and 0.2% of the cases in the registry, respectively. These three counties are ethnically diverse and are representative of the overall population of western Kenya.

One-day screening special events were conducted at public health facilities serving the three counties. In the week before screening, communities were mobilized to participate by local leaders who invited everyone in the nearby area to attend breast cancer screening events. On the advice of health center leaders, both men and women in the target communities were invited to attend to secure male support for the screening activity. We acted on this advice because Kenya is predominantly a paternal society in which women usually seek permission from men before attending events like screening, vaccination, and family planning. The screening events included education and CBE provided by trained physician oncologists. At the screening event, any participants with breast lumps detected by CBE underwent fine-needle aspiration immediately. Those with cytology reported as suspicious for malignancy underwent core needle biopsies 1 week later followed by appropriate treatment at MTRH.

### Study Design

The study was a cross-sectional survey conducted in two parts. Part one (survey of screening participants) involved administering a questionnaire to all consenting women at CBE events. For this survey, we used the validated breast module of the Breast Cancer Awareness Module (BCAM) survey.^15^ Prior work had been done to modify its language to improve the face validity and understandability in Kenya.^16^

The second part (survey of community women who did not volunteer) involved home-based interviews of women who had not presented themselves for screening and who were identified by a systematic random sampling of residents within a 5-km radius along all roads leading to the health center. This survey of community residents used the same BCAM survey and was conducted the day after the screening event. Ethical approval was obtained from the Moi University Institutional Research and Ethics Committee as well as the Indiana University Institutional Review Board.

### Study Procedures

The questionnaire was administered in one of two languages (English or Kiswahili) by trained research personnel. Written consent was obtained from participants. Questionnaire items included the six domains of BCAM: sociodemographic characteristics, socioeconomic characteristics, experience with previous breast examinations, prior training in how to examine for a breast lump, knowledge of availability of screening programs, and perceived barriers to CBE if a woman noted changes in her breast. An open-ended question on reasons for not attending the current screening event was asked only of those who had heard about the screening event but had not attended. Another open-ended question with structured prompts inquired about preferences for learning about future screening events.

### Data Analysis

Data analysis was performed by using STATA SE 13 (STATA, College Station, TX). Categorical variables were summarized as frequencies and the corresponding percentages, whereas continuous variables were summarized as medians and the corresponding interquartile ranges (IQRs). Gaussian assumptions were assessed empirically by using the Shapiro-Wilk test for normality. Association between categorical variables was assessed by using Pearson’s χ^2^ test, whereas association between a continuous variable and a binary variable was assessed by using Wilcoxon two-sample test (Mann-Whitney *U* test). A logistic regression model was used to assess the joint effect of the covariates on the outcome. The covariates that were associated with the outcome at the bivariate level were included in the multivariable level. Variables that had been established to be associated with and/or were known a priori to be associated with each other were not included together in the logistic model to avoid multicollinearity. Only the independent variable that was thought to be the most important and statistically significant in bivariate analyses was included. For the logistic model results, we report the odds ratios (ORs) and the corresponding 95% CIs. Age as used in the logistic regression model was scaled down by 10 years to be able to compare two persons who were 10 years apart. Only significant results were presented in the tables; the remaining data are available from the corresponding author upon request.

## RESULTS

A total of 1,511 volunteers (1,238 women and 273 men) attended CBE screening. After CBE, 594 women (48% of total screening attendees) consented and were interviewed by using the modified BCAM questionnaire. A total of 467 women who did not attend were interviewed in their homes. Men were not interviewed. The overall median age (Table 1) was 34 years (IQR, 26 to 44 years). More than three quarters of participants (809 [76%]) were married, and the rest were single, separated, divorced, or widowed. The median number of biologic children that participants had was three (IQR, two to five children), whereas the median number of siblings was seven (IQR, five to nine siblings). Half the participants had either no education or elementary education. Fifty-six percent (595) of the participants were unemployed.

**Table 1:**
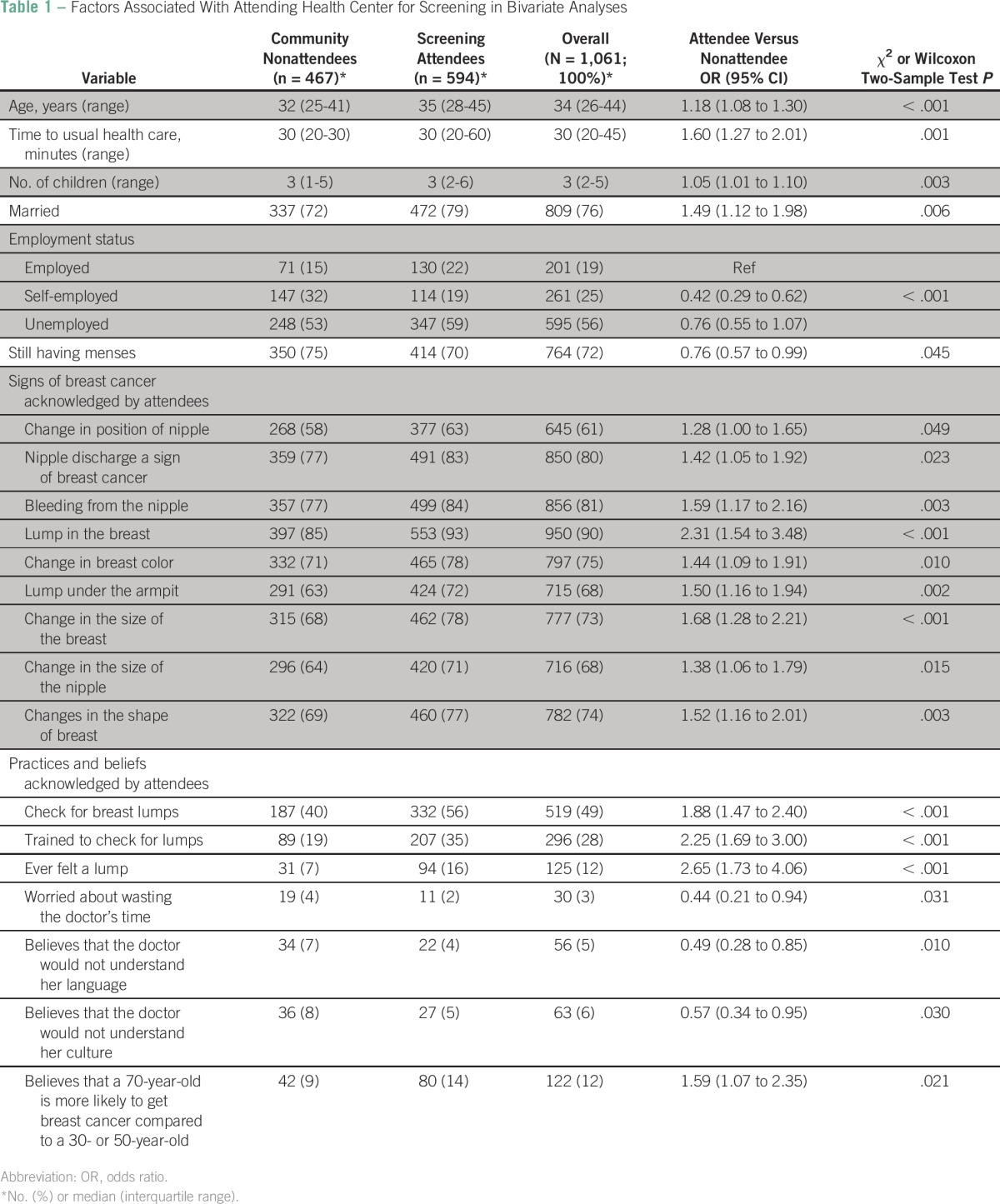
Factors Associated With Attending Health Center for Screening in Bivariate Analyses

As shown in Table 1, attendees were older, more often married, had more children, more often employed, and were more often menopausal. Attendees were more likely to validate signs and symptoms typical of late-stage breast cancer (change in position, enlargement, discharges, bleeding from nipple; breast lump; change in skin color; increase in size of lump; and lump in the armpit). Attendees also were more likely to report checking their breast for lumps (self-examination), having been trained in self-examination, and having ever felt a lump in their breasts. Attendees were also less likely than nonattendees to worry about wasting a doctor’s time, that the doctor might not understand their language or culture, and more often knew that older women were most likely to get breast cancer.

Table 2 shows the variables independently associated with uptake of breast cancer screening. Attendees were older, took a longer time to travel to the health facility, more often employed, believed that a lump in the breast is a sign of breast cancer, self-examined for lumps in the breast, had been previously trained to check for lumps, had ever felt a lump in the breast, were less worried about whether a doctor would not understand their language, and knew that older women are more likely to get breast cancer.

**Table 2:**
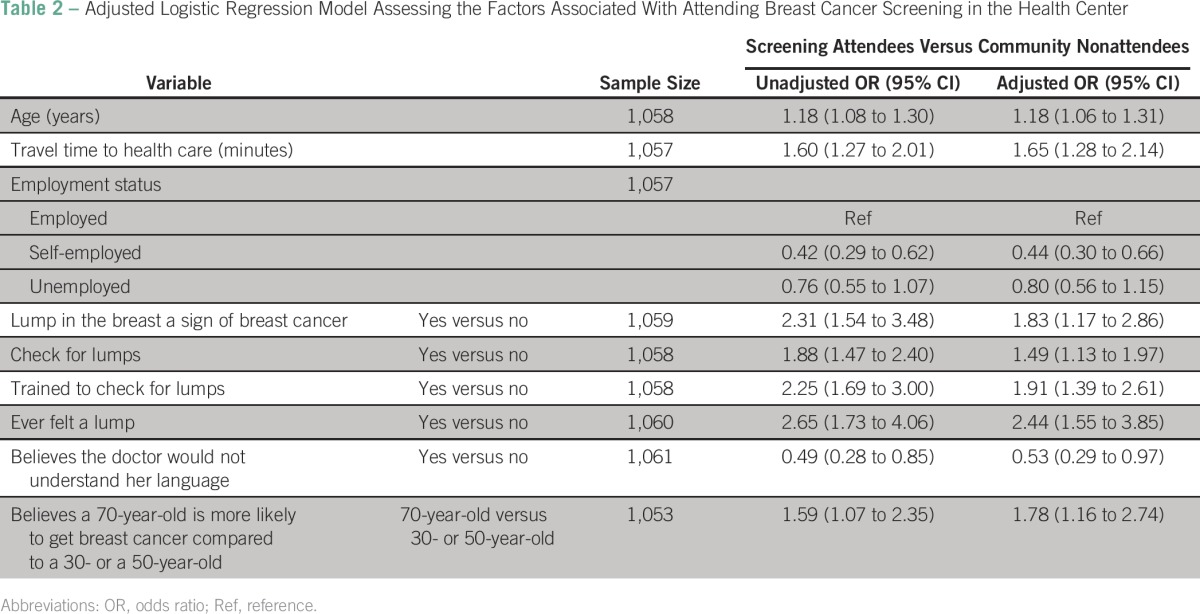
Adjusted Logistic Regression Model Assessing the Factors Associated With Attending Breast Cancer Screening in the Health Center

Because BSE showed a substantial association with attendance at CBE screening (an 88% increased chance of checking for lumps among those who visited the clinic for breast cancer screening compared with those who did not visit the clinic for breast cancer screening; odds ratio, 1.88; 95% CI, 1.47 to 2.40) within our pooled data set, we also explored factors associated with reported BSE. In bivariate analyses, many independent variables demonstrated significant associations with BSE (results available from the corresponding author upon request). The variables that were statistically significant in the bivariate analysis level were included in the multivariate level. The results of the final model are shown in Table 3. The adjusted effects showed that participants who reported BSE were younger, more often employed, had better education, reported family history of breast cancer, and believed that cancer presented as a mass in the breast. There was a strong association with reports of prior training for BSE, ever having felt a lump in her breast, and having had prior screening.

**Table 3:**
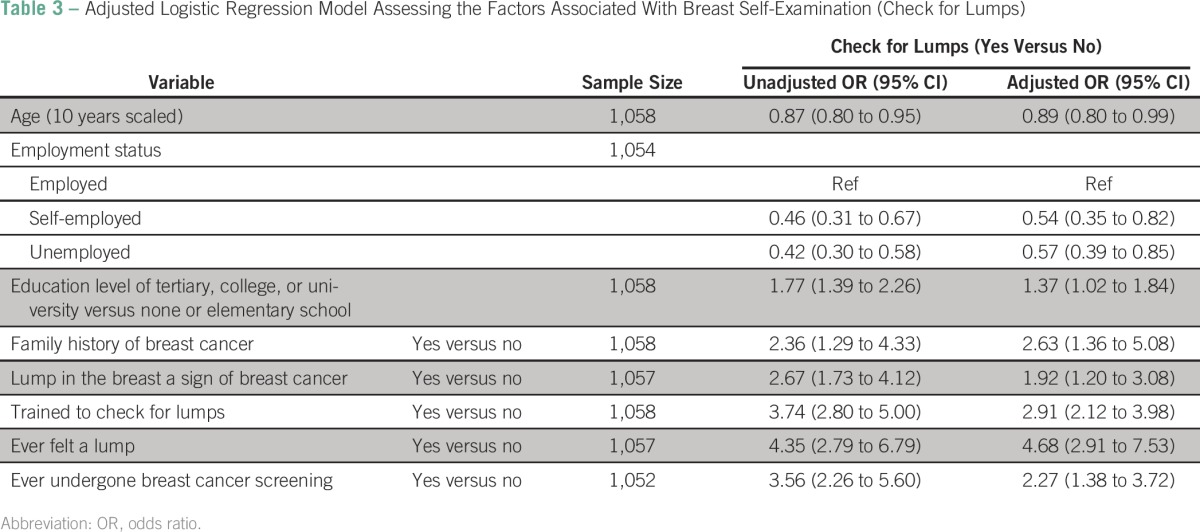
Adjusted Logistic Regression Model Assessing the Factors Associated With Breast Self-Examination (Check for Lumps)

### Clinical Yield of CBE Special Events

As noted previously and in Table 4, a total of 1,511 volunteers underwent CBE. A total of 59 breast abnormalities were detected, including lumps and ulcers (eight in men and 51 in women). Only one man of five with lumps consented to fine-needle aspiration (reported as negative for malignancy). Three other men had ulcerating disease presumed to be breast cancer. Thirty-seven women accepted fine-needle aspiration, and 15 were biopsied. Three men and four women had breast cancer confirmed by histology. All the men and two women with breast cancer had ulcerating lumps, believed their disease was advanced, and declined further care. One woman had stage III disease and underwent treatment at MTRH and Kenyatta National Hospital. The other woman was lost to follow-up.

**Table 4:**
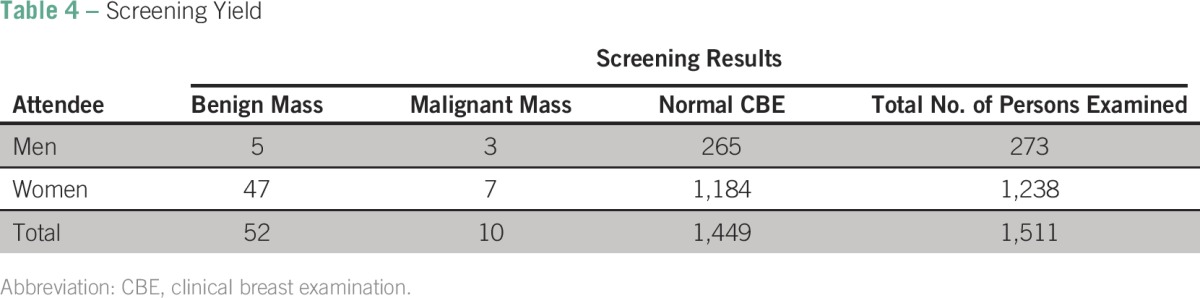
Screening Yield

### Interest in Future CBE Special Events

All the health center participants and 98% of community participants interviewed reported interest in participating in annual screening if such programs were available. For that reason, and because the Walther Project group wanted to know how best to announce future activities, we added a question to the BCAM survey that inquired about preferred mechanisms for alerting the communities (Table 5). Fifty-four percent (574) chose the local radio station as the best means of communication to help convey breast cancer information. The rest chose national radio (41%) and mailed information (5%).

**Table 5:**
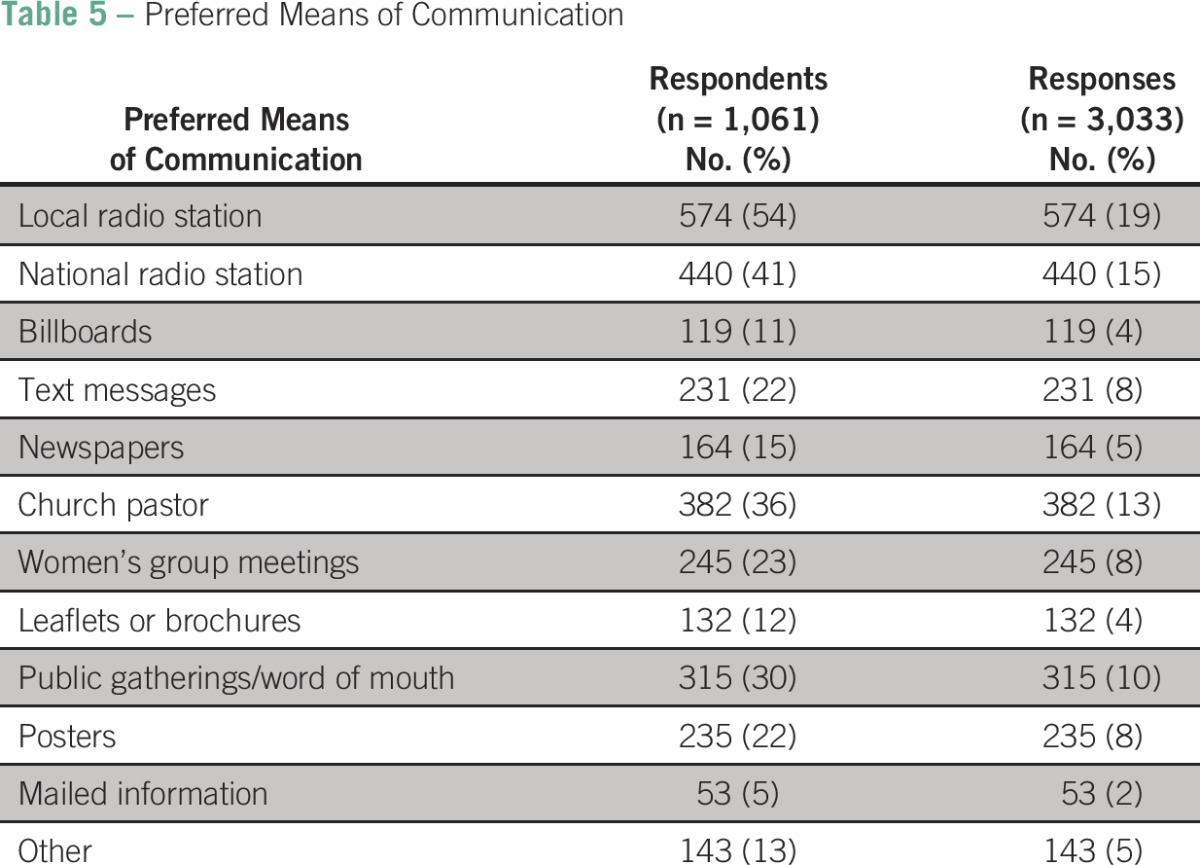
Preferred Means of Communication

## DISCUSSION

Mammographic screening of women for breast cancer followed by early diagnosis and intervention has been shown to decrease mortality from breast cancer in resource-rich countries.^17,18^ Unfortunately, mammography is generally not available in LMICs because of a lack of both human and physical resources.^19^ In its absence, CBE every 3 years for women younger than age 40 years and annually for women older than age 40 years has been used.^10,11^ CBE may be effective for detection in LMIC settings because the mean tumor size at presentation among women in these countries is palpable.^8^ A study in Sudan suggested that CBE using local volunteers can increase detection of breast cancer in asymptomatic women.^20^ Other studies conducted in India and Malaysia have suggested that CBE can significantly downstage breast cancer in screened populations. Follow-up of longer-term outcomes is ongoing to assess mortality benefit.^21,22^ In addition, CBE increases breast cancer awareness and early response to symptoms, a combination of effects that may have decreased mortality from breast cancer in the East Anglia study.^23^

In our circumstances, we adopted CBE as the best available option for screening. We focused on social mobilization for mass CBE screening as our primary approach, but another option might be integrating CBE into provision of primary health services at clinic appointments for other indications. At AMPATH Oncology Institute, both approaches are used and have been found to be complementary. Challenging logistic arrangements, substantial cost, and restricted access to special events for women on particular days make mass screening a less-than-ideal approach to reach our populations. Our target study population, for example, was 45,187 women older than age 45 years residing in the catchment areas of the three rural health facilities per the last National Census in 1999. Although all were invited, only 1,238 women attended our special events. To increase participation in screening, a sustained awareness program and availability of screening services is required. Such a program can be integrated into primary care visits with other age- and sex-appropriate health risk screening services, such as those for hypertension and cervical cancer as practiced in the National Breast and Cervical Cancer Early Detection Program in America.^24^

Alternative approaches to create awareness of breast health and screening options are also needed. We noted that more than half of those surveyed preferred to be alerted about screening activities through messages broadcast by local radio stations. Health staff partnering with local radio stations to create public service announcements or participating in talk shows about breast health might promote and improve responsiveness to breast cancer screening opportunities.

### Was CBE Screening in Our Health Center-Based Approach Productive?

We examined 1,511 people (men and women) and diagnosed seven breast cancers, a yield of one breast cancer detected for every 215 people screened. This yield is lower than the one breast cancer for every 42 women screened by Luyeye Mvila et al^13^ in the Democratic Republic of Congo but higher than one for every 606 screened by Abuidris et al^20^ in Sudan. The higher yield in the study by Luyeye Mvila et al could be the result of the combined use of CBE and mammography, whereas Abuidris et al used CBE alone. There also seemed to be an educational product from the exposure of women to CBE. After being examined, women who attended CBE screening were 2.25 times more likely to report that they had been trained to do breast self-examination. The US Preventive Services Task Force recommends against teaching women BSE because of lack of mortality benefit and increased unnecessary biopsies,^7^ but we found that women who reported doing BSE were more likely to volunteer for CBE (56%) than those who did not (40%). From our data, there seems to be a relationship between BSE, training for BSE, and volunteering to be screened. Although we did not directly test for this effect, it is possible that exposure to CBE was interpreted by women as training on BSE. Because of this association, we at AOI are inclined to advocate for teaching BSE to women as in the National Guidelines for Cancer Management in Kenya.^9^

### Application of the Study Findings

The study has identified some of the factors associated with women who volunteer for breast cancer screening. These include being trained to feel for lumps, believing that a doctor would understand their language, and not being worried about wasting the doctor’s time. All these factors should be considered when designing breast cancer screening invitation messages. We have also developed a brief educational process that has been used to teach women more about breast cancer and CBE screening.^25^

The strengths of this study included the use (after modification for language and culture) of the BCAM survey, an internationally validated tool for measuring breast cancer awareness in various domains. The study participants were ethnically diverse and represented several Western Kenyan ethnic communities. The weaknesses of the study included a less-than-ideal participation rate in our health center-based survey and lack of mammography to confirm that women were reassured that they actually had normal breasts. Finally, we acknowledge that this study of screening may, like studies in other LMICs, be describing the characteristics and beliefs of women who had symptoms that led them to participate in the CBE special event in the first instance. In that sense, our special events may not have been true screening of asymptomatic individuals in a strict sense. Nonetheless, because the effort in our setting is to detect the presence of breast cancer at an earlier and less anatomically advanced stage, the activities we report may fairly be said to represent screening in the context of resource-scarce Kenya at this point in history.

Ninety-nine percent of all the women who participated in this study were willing to take part in future screening events. There is an opportunity to significantly increase breast cancer awareness in our communities by continuing to offer CBE during special events, integrating this service into primary care for those unable to participate in mass screening, disseminating breast health information to our communities, and actively educating and training those who present themselves for screening.
